# Draft Genome Sequence of Naganishia liquefaciens Strain N6, Isolated from the Japan Trench

**DOI:** 10.1128/MRA.00827-20

**Published:** 2020-11-19

**Authors:** Yong-Woon Han, Rei Kajitani, Hiroya Morimoto, Maierdan Palihati, Yumiko Kurokawa, Rie Ryusui, Bilge Argunhan, Hideo Tsubouchi, Fumiyoshi Abe, Susumu Kajiwara, Hiroshi Iwasaki, Takehiko Itoh

**Affiliations:** a School of Life Science and Technology, Tokyo Institute of Technology, Tokyo, Japan; b Institute of Innovative Research, Tokyo Institute of Technology, Yokohama, Kanagawa, Japan; c College of Science and Engineering, Aoyama Gakuin University, Sagamihara, Kanagawa, Japan; Vanderbilt University

## Abstract

The draft genome sequence of the deep-sea yeast *Naganishia liquefaciens* strain N6, isolated from the Japan Trench, is reported here. This strain was previously classified into a *Cryptococcus* clade. Phylogenetic analysis using the presented sequence suggests that strain N6 is in the clade of the genus *Naganishia*.

## ANNOUNCEMENT

Naganishia liquefaciens (phylum Basidiomycota, class Tremellomycetes) strain N6 was isolated from the Japan Trench (6,500 m) (1, 2). Sediment samples were spread onto yeast extract-peptone-dextrose (YPD) plates (3) containing penicillin and streptomycin sulfate, and colonies were isolated. Although initially named *Cryptococcus liquefaciens* based on a comparison of the 18S rDNA with that of Cryptococcus albidus (1, 2), Cryptococcus albidus was subsequently assigned to the genus *Naganishia*; *Cryptococcus liquefaciens* was therefore renamed *Naganishia liquefaciens* (4, 5). Strain N6 is tolerant to heavy metals (1, 2, 6). Many deep-sea microorganisms have evolved to survive under extreme conditions, and their characterization is potentially crucial for the production of useful biomolecules.

Strain N6 was cultivated on YPD medium (3) at 30°C, and genomic DNA was prepared (Dr. GenTLE kit, TaKaRa Bio). Whole-genome sequencing was performed using the Illumina MiSeq platform. Three paired-end (kit, TruSeq; 34,806,922 reads; read length, 150 or 300 bp; insert sizes, 400 to 550 bp) and three mate pair (kit, Nextera mate pair; 25,211,788 reads; read length, 250 bp; insert sizes, 4,000 to 10,000 bp) libraries were generated (total, 14.7 Gbp). Default parameters were used except where otherwise noted. The reads were trimmed using Platanus_trim v1.0.2 (http://platanus.bio.titech.ac.jp/pltanus_trim). *De novo* assembly was performed by Platanus v1.2.1 (7), inputting all but the 10-kbp mate pair libraries. Misassemblies were corrected based on the physical coverage of the 10-kbp mate pairs. Some gaps were filled by additional Sanger sequencing of the PCR products of gap-flanking primers (44 reads; SRA accession number DRR244395; alignment tool, BLASTN). The complete mitochondrial genome (GenBank accession number BLZA01000059.1) was constructed using Platanus (“assemble -n 200” command). The numbers of resulting scaffolds and contigs, the total length, the scaffold *N*_50_ value, the contig *N*_50_ value, the gap rate, and the GC content were 59, 87, 19.44 Mbp, 1.03 Mbp, 0.62 Mbp, 0.10%, and 53.38%, respectively.

To determine strain N6’s gene structure, total RNA from cells grown in YPD plus adenine (YPAD) or YPAD containing 10 mM CuSO_4_ was prepared (Nucleospin RNA kit, Macherey‐Nagel). Four transcriptome sequencing (RNA-seq) libraries (two replicates for each condition) were prepared [TruSeq kit with poly(A) selection], and RNA-seq was performed using an Illumina MiSeq instrument (read length, 300 bp), resulting in 37,287,236 reads (total 8.10 Gbp). The protein-coding gene structure on the scaffolds was predicted using FunGAP v1.0.0 (8) and proteins from Cryptococcus neoformans and Cryptococcus gattii (GenBank accession numbers GCA_000091045.1 and GCA_000185945.1, respectively). A total of 6,883 genes were predicted. The average lengths of transcripts and coding DNA sequences (CDS) were 1,999 bp and 1,621 bp, respectively. In addition, 38,748 introns were identified, with 93.7% of genes containing at least one intron.

**FIG 1:**
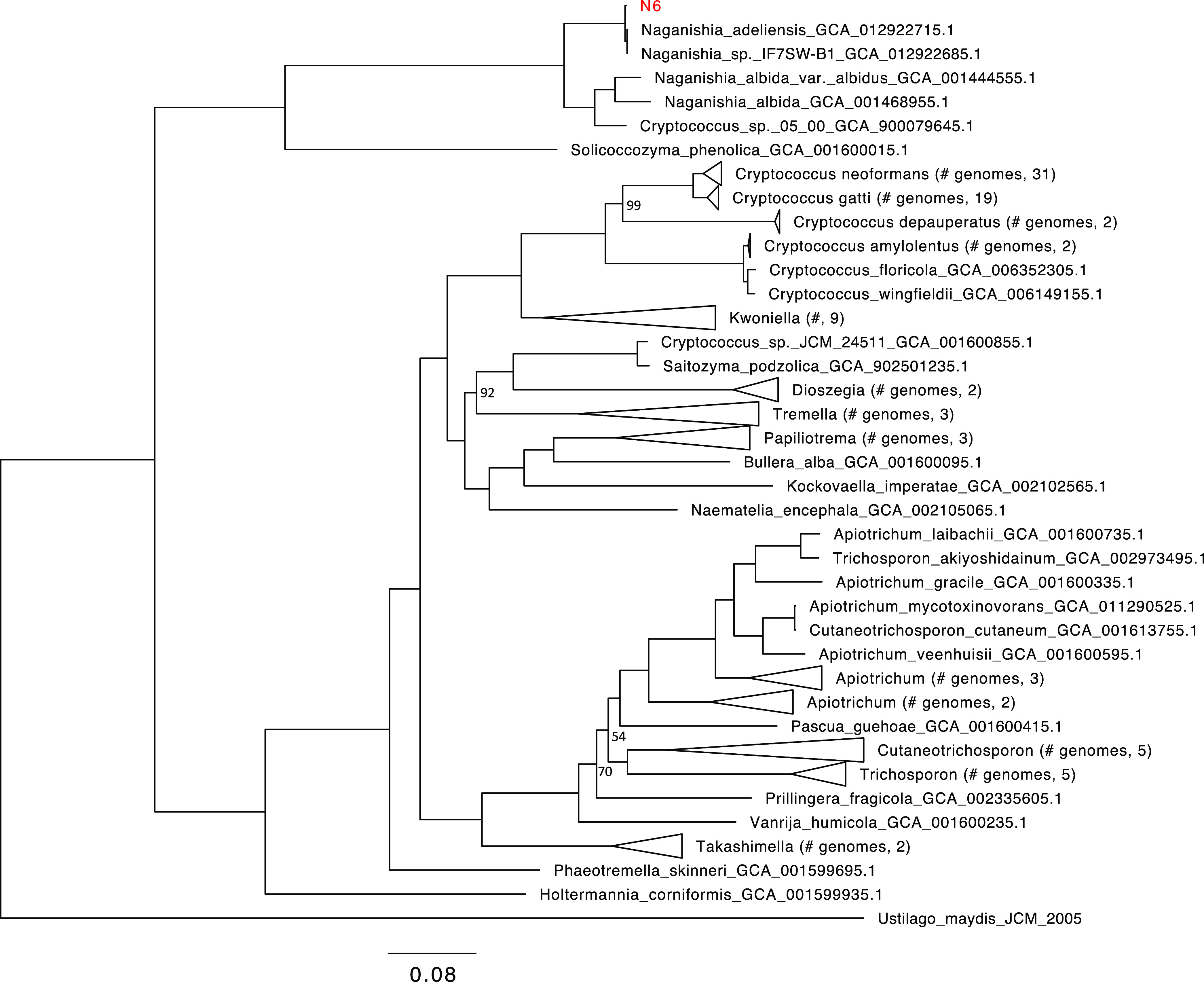
Phylogenetic tree of genomes in the class *Tremellomycetes*. *N. liquefaciens* strain N6 is shown in red. Ustilago maydis JCM 2005 is used as an outgroup. The number of bootstrap replicates is 1,000, and the percentages of bootstrap supports (1% to 100%) are shown as the numbers near the nodes (only the bootstrap supports of <100 are displayed). Branch lengths are based on the number of substitutions per site, as indicated by the scale bar at the bottom. The number of sites in the multiple alignment is 133,605 (amino acids).

We downloaded 113 genomes in the class Tremellomycetes and the genome of Ustilago maydis JCM 2005 as an outgroup from the GenBank database. Single-copy orthologs were identified using BUSCO v4.0.6 (9) with the basidiomycota_odb10 data set (1,764 orthologs). In the strain N6 genome, 1,613 (91.3%) benchmarking universal single-copy ortholog (BUSCO) complete genes were detected. For each ortholog group, protein sequences were aligned using MAFFT v7.455 (10), and gaps were removed. From the concatenated alignment, a maximum-likelihood tree was reconstructed using IQ-TREE v1.6.12 (11) with the LG+I+G4+F model and 1,000 bootstrap replicates. The resulting tree (Fig. 1) suggests that strain N6 belongs to the genus *Naganishia*.

### Data availability.

The raw reads and the draft genome have been deposited in DDBJ/ENA/GenBank under BioProject accession number PRJDB10172 and the whole-genome shotgun project number BLZA00000000.1, respectively. The SRA accession numbers for paired-end reads are DRR237062, DRR237063, and DRR237064. The SRA accession numbers for mate pair reads are DRR237065, DRR237066, and DRR237067. The SRA accession numbers for RNA-seq reads are DRR237068, DRR237069, DRR237070, and DRR237071. The SRA accession number for Sanger reads is DRR244395.
